# Towards a First-Person Perspective Mixed Reality Guidance System for Needle Interventions

**DOI:** 10.3390/jimaging8010007

**Published:** 2022-01-07

**Authors:** Leah Groves, Natalie Li, Terry M. Peters, Elvis C. S. Chen

**Affiliations:** 1 School of Biomedical Engineering, Western University, London, ON N6A 3K7, Canada; nli253@uwo.ca (N.L.); tpeters@robarts.ca (T.M.P.); chene@robarts.ca (E.C.S.C.); 2 Robarts Research Institute, Western University, London, ON N6A 5K8, Canada

**Keywords:** surgical navigation, ultrasound, image guided intervention, mixed reality systems, quantitative skills assessment, central venous catheterization

## Abstract

While ultrasound (US) guidance has been used during central venous catheterization to reduce complications, including the puncturing of arteries, the rate of such problems remains non-negligible. To further reduce complication rates, mixed-reality systems have been proposed as part of the user interface for such procedures. We demonstrate the use of a surgical navigation system that renders a calibrated US image, and the needle and its trajectory, in a common frame of reference. We compare the effectiveness of this system, whereby images are rendered on a planar monitor and within a head-mounted display (HMD), to the standard-of-care US-only approach, via a phantom-based user study that recruited 31 expert clinicians and 20 medical students. These users performed needle-insertions into a phantom under the three modes of visualization. The success rates were significantly improved under HMD-guidance as compared to US-guidance, for both expert clinicians (94% vs. 70%) and medical students (70% vs. 25%). Users more consistently positioned their needle closer to the center of the vessel’s lumen under HMD-guidance compared to US-guidance. The performance of the clinicians when interacting with this monitor system was comparable to using US-only guidance, with no significant difference being observed across any metrics. The results suggest that the use of an HMD to align the clinician’s visual and motor fields promotes successful needle guidance, highlighting the importance of continued HMD-guidance research.

## 1. Introduction

Central venous catheterization (CVC) is a routine procedure in intensive care medicine and anesthesiology, performed over 6 million times annually in the United States and Europe [1,2]. The internal jugular vein (IJV) is the most utilized insertion site [3], with clinical indications including administration of medication, recording of central venous pressure or other haemodynamic parameters [4], haemodialysis [5], and accessing the right atrium [6]. Traditionally, CVC placement via the IJV is performed using landmark techniques based on knowledge of anatomic structures and palpation of the carotid artery (CA) [7,8]. However, this approach does not account for anatomic variations or the presence of venous thrombosis at the CVC insertion site [7,8,9], resulting in accidental puncture of the CA with incidence of up to 9.4% [1] and leading to devastating events including hematoma, stroke and death [10,11].

Interpatient anatomic variations [12,13] and the presence of venous thrombosis can generally not be identified using a landmark technique. However, ultrasound (US) can be used to visualize anatomic structures and confirm patency of the vein to help avoid unintended arterial puncture or unsuccessful cannulation [8]. The ability of US-guidance to reduce the number of complications and to increase the safety and quality of CVC placement has been demonstrated in several systematic reviews and meta-analyses [7,8]. The use of real-time US for guiding the puncture of the vein and confirming the correct needle, wire and catheter position in the vein is now the recommended approach endorsed by many professional societies [9,14,15,16].

The US images used for needle guidance can be captured with the US probe oriented transversely to the vein, showing the short axis (SAX) image such that the needle is inserted “out-of-plane”, or parallel to the vein [14,17]. Alternatively, the long axis (LAX) can be captured such that the needle is inserted “in-plane”, or at an oblique orientation relative to the US image [14,17]. While there are no data to suggest which approach is superior [8], SAX with the out-of-plane approach is the preferred option [16] because it is easier to learn [18] and results in higher success rates with the first attempt for CVC placement in the IJV [19]. However, in this approach, the needle tip and shaft both appear as hyperechoic spots in the US image, making it challenging to distinguish one from the other. The uncertainty surrounding the exact location of the needle tip in US images is associated with inadvertent arterial, posterior IJV, and pleural punctures [11,20,21]. As a result, acute adverse events still occur under US-guided CVC, with an adverse event rate as high as 19.7% being reported in the literature [22] (or a complication rate of 4.6% as reported by [23]). The key challenge for US-guided CVC is maintaining visibility of the needle tip in the US image throughout the entire insertion [8]. The success of vein puncture may be affected by the inability to visualize all relevant anatomy and the needle tip within the limited 2D US field of view (FoV). Additionally, the clinician obtains the visual information to guide the insertion from the US console while simultaneously performing the insertion on the patient. This results in a decoupling of their visual and motor fields, which may affect insertion success.

To address these limitations of US-guided CVC, several mixed-reality (MR) systems were developed to provide 3D visualizations for needle-insertion. Here, the term MR is employed to refer to visualization techniques that fall on the reality-virtuality spectrum between fully real and fully virtual environments [24]. Rochlen, Levin, and Tait developed a training system for central-line insertion using augmented reality (AR) glasses [25], the use of which allows participants to place the needle in the mannequin using a “first-person” point-of-view AR system, where the relevant internal anatomical landmarks are rendered within the AR glasses and perceived by the users in their anatomically correct positions. While the technical details were lacking in [25], users participating in their study (n=40) reported the first-person perspective AR view of the internal anatomy was helpful in guiding needle-insertion. Huang et al. [26] also introduced an AR-glass system for central-line simulation and teaching. Distinct from [25], this system displayed a concise CVC instructional slideshow as a checklist for participants to follow as they carried out the procedure instead of displaying the underlying anatomical structure as a visual aid. As these two systems were designed for teaching and training purposes, feasibility of clinical translation of these systems was not demonstrated.

**Figure 1:**
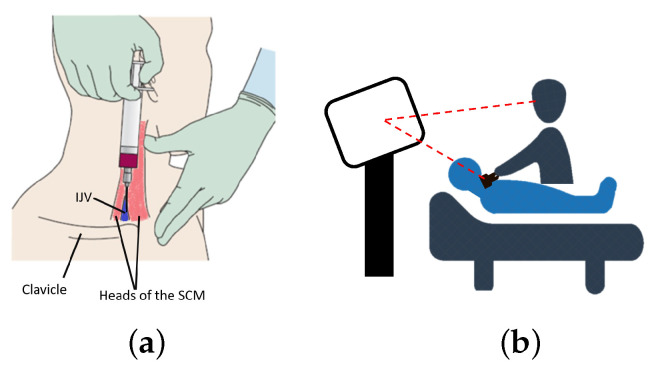
Comparison of guidance technique for CVC: (**a**) anatomical landmarks, (**b**) US-only guidance.

Ameri et al. [27] developed a surgical navigation system for guiding CVC puncture that employed a magnetic tracking system (MTS) to render virtual representations of the US transducer, surgical needle, and needle trajectory using a standard 2D monitor. Their visualization comprised the real-time position and orientation (pose) of the tracked needle relative to a stationary, front-facing US image to maintain consistency with the traditional US-guided procedure (Figure 1b). This system did not result in a significant reduction of the complications associated with the needle-insertion when compared to that experienced by expert users employing the US-only technique [27]. The performance of this visualization paradigm may have been influenced by both the fixed front-facing US visualization, as the user still relied on a 2D visualization to perform a 3D procedure, and the visual and motor disparity produced when the visualization device is exterior to the visual field of the phantom where the needle-insertion is performed.

In this paper, we investigate the efficacy of a first-person immersive MR system for the navigation of needles for central venous catheterization (CVC). While the long-term goal of this work is clinical deployment, we first must understand how the visualization paradigm associated with surgical information affects the complication rate during US-guided CVC. Similar to [27], we propose a needle guidance system using an MTS to provide, in real-time, the spatial relationship between surgical instruments relative to the patient anatomy. We compare the standard-of-care US-only guidance to the use of our MR guidance system, where the images are displayed both on a 2D monitor and also using a first-person perspective view available with a head-mounted display (HMD). We hypothesize that employing an HMD for visualization will improve the success of the needle-insertions compared to those that use US-only guidance or a conventional 2D monitor. Via a user study involving 31 clinicians and 20 medical students, this work highlights the importance of providing coherent visual and motor fields for image-guided surgical applications.

## 2. Materials and Methods

To evaluate the efficacy of various visualization paradigms, we developed a simulated CVC experimental setup in a controlled laboratory setting. Our system comprises an anthropomorphic phantom, a clinical US scanner, and a surgical navigation system coupled with display technologies.

### 2.1. Anthropomorphic Phantom

**Figure 2:**
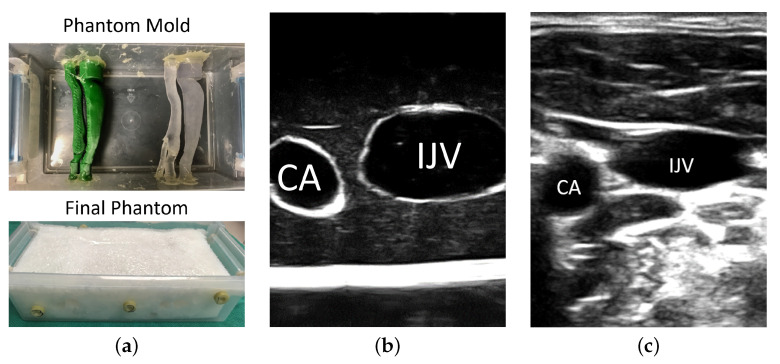
Anthropomorphic Phantom for simulated CVC procedures: (**a**) positive mold of the neck vasculature was embedded into an US-compatible tissue-mimicking material (PVAc) to create a wall-less hollow structure, (**b**) SAX view of the phantom depicting the simulated CA and IJV, and (**c**) SAX view of human neck vessels as an example of vascular anatomy under ultrasound courtesy of a healthy volunteer.

A US-compatible phantom for simulated CVC insertion was created, consisting of two hollow vasculature structures representative of the CA and IJV. The CA and IJV were segmented in 3D from a CT scan of a subject’s neck, with the resulting volumes used to manufacture molds using a 3D printer. These molds were then embedded as cores with polyvinyl alcohol cryogel (PVAc). The vessels were positioned such that the IJV was 1.5 cm from the surface of the phantom, which is representative of a normal case. After two freeze-thaw cycles, the core was removed from polymerized PVAc, creating hollow and wall-less vascular structures that mimic the appearance of the CA and IJV under US imaging (Figure 2). Polymerized PVAc after two freeze-thaw cycles exhibits speed-of-sound (SoS) of 1540 m s^−1^ and a tactile sensation similar to that of human tissue [28,29]. The mechanical properties of PVAc after two freeze-thaw cycles closely match that of a vessel wall [30]. Additionally, clinicians from a wide range of disciplines, including anesthesiology, cardiology, and neurology, as well as the participants in this study, have commented that interaction with PVAc models feels natural and replicates the tactile sensation of human tissue. While we have not measured the insertion forces explicitly, the study participants commented that the resistance to insertion and force required to insert the needle closely mimic the in vivo situation. To simulate pose tracking of the patient anatomy, we placed this phantom in a plastic container that contained 8 hemispherical fiducials markers and a 6 Degree-of-Freedom (DoF) magnetic pose sensor. A cone-beam CT scan of this phantom was taken (O-arm, Medtronic, Minneapolis, MN, USA), from which the simulated vasculature and hemispherical fiducials were segmented, serving as ground truth. The segmented locations of these hemispherical fiducials were used to register the CT volume, and hence the segmented vasculature, with the MTS using a least-squares solution [31,32]. This phantom serves as a surrogate for patient anatomy, producing US images where the CA and IJV can be easily interpreted by the user (Figure 2).

### 2.2. Spatial Calibration for Tracked Apparatuses

**Figure 3:**
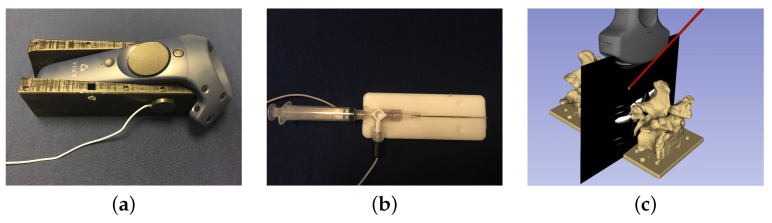
(**a**)The co-calibration apparatus tracked by the VIVE controller and magnetic pose sensor; (**b**) the calibration apparatus for the syringe; and (**c**) visual representation of an example of tracked tools registered in the HMD’s coordinate system, where the alignment between the models of the spine and needle and their reflections in the US image indicated the total system accuracy.

The surgical guidance system comprises either a conventional 2D monitor or a MR HMD display (HTC VIVE Pro, HTC, New Taipei City, Taiwan), a magnetic tracking system (Aurora, NDI, Waterloo, ON, Canada), a clinical US scanner (SonixTouch, BK Medical, Peabody, MA, USA), and a surgical hypodermic (7 cm metallic needle with a 10 mL syringe (Figure 3b). Real-time US imaging of the phantom, at an imaging depth of 6 cm, was achieved via a linear transducer (L14-5, BK Medical, Richmond, BC, Canada). A 6-DoF magnetic pose sensor was rigidly attached to the US transducer, with the geometrical relationship between the US image coordinate system and the magnetic pose sensor calibrated using a Procrustean point-to-line registration algorithm [33]. To track a surgical hypodermic needle, a 6-DoF magnetic pose sensor was integrated into a 3-way Luer-lock connector, used to connect the metallic needle with the plastic syringe. A tracked “template” with an exact negative imprint of the needle assembly was used to calibrate the spatial pose of the needle assembly with respect to its magnetic pose sensor (Figure 3b). Six hemispherical divots were embedded into this template, allowing the known geometry of the negative imprint of the needle assembly to be registered with respect to its magnetic pose sensor [34].

The HTC VIVE Pro is a video-pass-through (VPT) HMD, selected for its high-resolution display and visual fidelity. The VIVE Pro HMD and its hand-held controller are spatially tracked by a laser-based approach known as the “Lighthouse”system, where two base-stations (light houses) emit IR light into the FoV. The pose of the HTC VIVE components is obtained based on the interaction between the the IR light and the reflective markers on the devices. This tracking information is supplemented using internal inertial motion sensors. To integrate the HTC VIVE Pro as a visualization device, the Lighthouse tracking system must be co-registered with the MTS. Using the principle of template-based calibration [34], we developed a co-registration apparatus comprising a magnetic 6-DoF pose sensor and the negative imprint of a HTC VIVE Pro hand-held controller (Figure 3a). This apparatus has a set of embedded hemi-spherical divots at known locations relative to the negative imprint, allowing for the geometry of the hand-held controller to be registered with respect to its magnetic pose sensor. After an HTC VIVE Pro controller is inserted into this template, the simultaneous tracking of this apparatus provides the co-registration between the MTS and the HTC Lighthouse trackers [35]. The accuracy of this co-registered hybrid tracking system was previously validated, exhibiting a positional trueness and precision of (0.48±0.23) mm, and a rotational error of (0.64±0.05) deg, respectively [35]. The accuracy of the overall system is displayed in Figure 3c.

### 2.3. Visualization Paradigm

**Figure 4:**
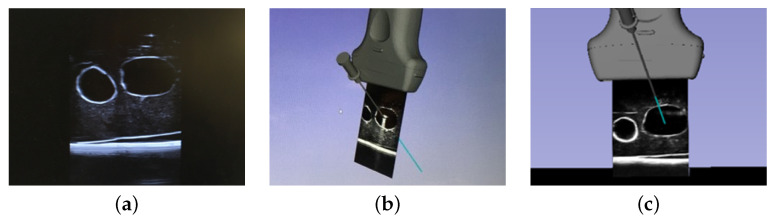
Visual representations of each mode of visualization where (**a**) is the US-only system, (**b**) is the monitor system, and (**c**) is the HMD system. Images (**b**,**c**) comprise models of the US probe, needle, needle trajectory, and the calibrated US image.

**Figure 5:**
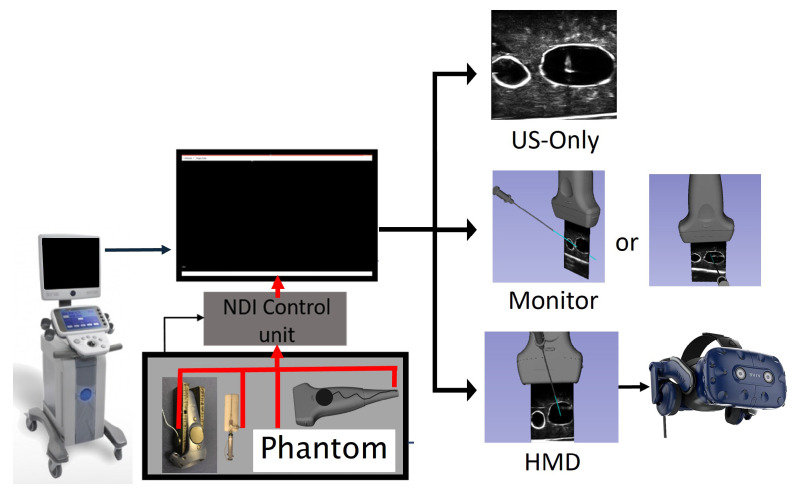
Experimental setup illustrating the hardware used, including the tracking and HMD components, as well as the three modes of visualization.

Three visualization paradigms were evaluated: (a) the direct visualization of a US image on the US console, which is the standard of clinical practice; (b) a 3D MR visualization displayed on a 2D monitor; and (c) a 3D MR visualization displayed inside an HMD, as depicted in Figure 4. In both 3D MR visualization paradigms, shown either using a 2D monitor or an HMD, virtual representations of the needle and tracked probe, as well as a representation of the needle trajectory (a 10 cm blue extension from the needle tip) were depicted on the display. The experimental setup, including the hardware and visualizations, is provided in Figure 5. This figure depicts the HTC VIVE Pro HMD and two sample graphics of the visualization and perspective used during the monitor case.

### 2.4. Participants

Two groups of participants were recruited for this study. The “expert” group comprised 31 clinicians who have been trained in US-guided central-line insertion techniques and have performed at least 15 clinical insertions. These participants are considered as experts, since current literature suggests that a user becomes proficient in US-guided CVC after 8 clinical insertions [36]. Members of the expert cohort have on average performed 319 clinical US-guided insertions with an average of 5.5±3.3 years of experience. The second group consisted of 20 medical students who did not have prior training or experience with US-guided CVC but were knowledgeable in neck vascular anatomy. All participants were recruited with written consent according to our local REB regulation (Western University REB 107254). The order of modes of visualization was randomized and assigned to participants in a counter-balanced manner, such that there was an equal number of participants assigned to each ordered sequence.

Prior to the involvement in this study, each participant was instructed with respect to the needle-insertion required for the simulated CVC using the neck phantom. This briefing was scripted to provide a consistent explanation of the experiment for each participant. The explanation included an introduction to the phantom, such that participants understood how to identify the IJV; an overview of the three modes of visualization they would use; and their goal, which was defined as positioning the needle at the center of the IJV. The vessel on the left-side of the phantom (Figure 2) was used as an example to train the users for all visualization modes. Participants were given time to perform needle-insertion using the standard-of-care US-only mode, as well as using MR with a 2D and HMD displays, in their assigned order, until they were comfortable with the experimental setup using the different modes of visualization. The study was then conducted using the vasculature on the right-side, whose geometry differed from that on the left. Each participant executed a needle-insertion into the right-hand side vessel for each mode of visualization. They were allowed sufficient time between switching the visualization modes, to rest and adapt to the new environment, ensuring that the recorded data were not skewed by the participant’s memory of the spatial orientation of the phantom. The streaming US video, execution time, and pose of the tracked apparatus were recorded for subsequent analysis. Following the study, users completed a questionnaire pertaining to their experience with the guidance system.

### 2.5. Data Analysis

The recorded pose data for all tracked components were processed to extract (i) the procedure time and (ii) accuracy metrics to evaluate user performance under each mode of visualization. We measured the procedure time from when the needle was positioned on the surface of the phantom until the user completed the insertion. Three metrics were used to analyze the accuracy of needle-insertion. First, insertion success was defined as a binary metric where an insertion was deemed successful if the final needle tip location was inside the target IJV. In addition, two distance-based metrics were calculated: the unsigned distance between the final needle tip position to the center line of the vessel and the signed distance between the final needle tip position to the closest point on the vessel wall. The signed distance was chosen for the latter, being negative if the final need tip position was outside of the target IJV.

**Figure 6:**
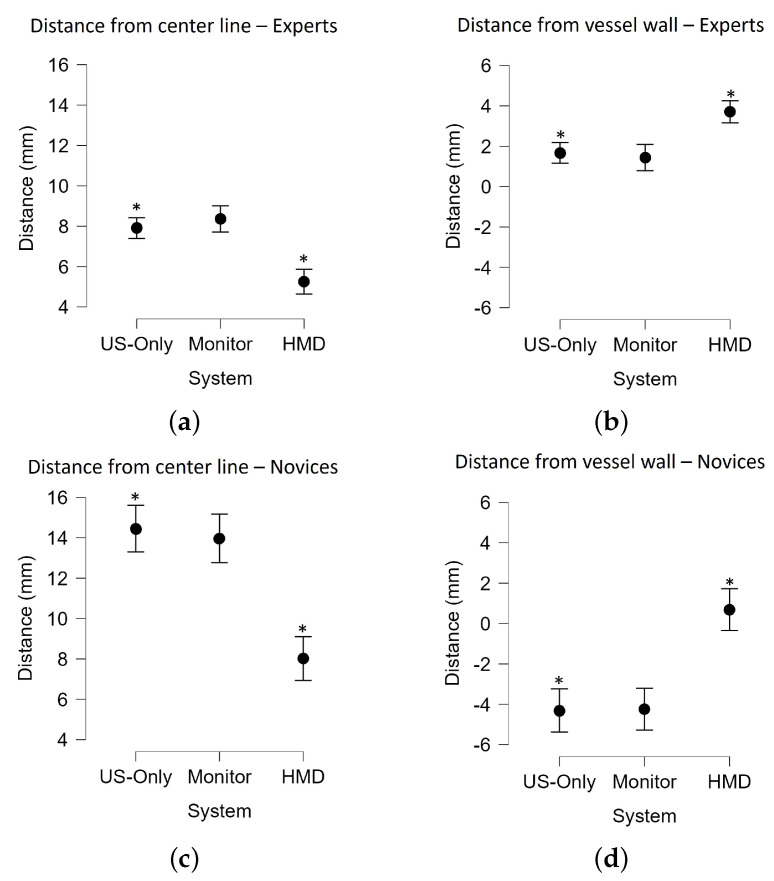
(**a**) Distance metrics presented with respect to system where (**a**,**c**) summarize the distances from the final needle tip position to the closest point on the center line of the vessel for experts and novices, respectively, and (**b**,**d**) summarize the distances from the final needle tip position to the closest point on the vessel wall for experts and novices, respectively. Metrics with significant differences between systems (*p* < 0.05) are denoted with *.

Results derived from continuous metrics, such as time and distance, were found to be normally distributed, as demonstrated by the Kolmogorov–Smirnov test prior to further statistical analysis. These continuous metrics were then compared using a repeated measures ANOVA across the three modes of visualization: US-only, 2D monitor, and HMD. Metrics that returned a *p*-value less than 0.05 from the ANOVA analysis underwent a least-squares distance multi-comparison post-test to compare between each pair of modes, as reported in Table 1 and Table 2. This test returns six possible comparisons between the metric being analyzed for the US-only, 2D monitor, and HMD results. Results of these tests with statistical significance (p<0.05) are summarized in Figure 6.

The success rate defined by the percentage of insertions that were successful is a discrete metric; therefore, the Chi-squared test was performed. For this test, a *p*-value of less than 0.05 indicated that the frequency rates in the contingency table were significantly different across the group. The success rates and p-values less than 0.05 are denoted in Table 3. The user questionnaire results were converted from the continuous scale to numerical values and are summarized in Table 5. While the time and number of CA punctures were not found to have any significant difference between systems, they are nonetheless summarized in Table 4.

The questionnaire required responses within a continuous scale where the center and either end were anchored with written descriptions, allowing the user to use the full scale range. These questionnaire responses were then converted into a numeric scale of between 1.0 and 10.0 (Table 5).

## 3. Results

The experimental results demonstrate that the use of the HMD as a first-person immersive MR visualization system significantly improved the number of successful CVC insertions and the targeting accuracy of these insertions for both expert clinicians and novice medical students. Ninety-four percent (93.5% or 29/31) of expert clinicians performed successful IJV insertion using the HMD system, an improvement from only 67.7% (21/31) by this same cohort using US-only guidance. The same trend was observed from novice medical students: seventy percent (70% or 14/20) of medical students successfully performed IJV insertion using the HMD system, compared to the success rate of 25% when using US-only guidance (Table 3).

The HMD also enabled these participants to perform more consistent targeting of the vessel when compared to the US-only mode of visualization. For both the expert clinicians and medical students, our results showed that the distance between the final needle position to the center line of the IJV was decreased from (7.9±4.5) mm to (5.4±2.3) mm when expert clinicians used the HMD for guidance instead of using US-only guidance. The same trend was observed for novice medical students as well with the average distance of the final needle position from the vessel wall decreasing from (14.5±7.1) mm for US-only guidance to (8.0±4.2) mm for HMD guidance.

## 4. Discussion

For the 2D monitor-based MR guidance, our data showed there was no significant improvement in targeting accuracy over US-only guidance for either the expert clinicians or the novice medical students. In our monitor-based MR guidance system, the virtual representation of a tracked needle, needle trajectory, US transducer, and streaming US video was visualized in a common coordinate system (Figure 4). The angle of view for US video was unconstrained, i.e., participants were free to adjust the vantage point, with most participants preferring an oblique viewing angle for improved 3D perception. These findings are consistent with the conclusions of Ameri et al. [27], who also found no significant improvements of the expert cohort when monitor-based MR guidance was used compared to the US-only mode of visualization. The key difference between their system and ours is that theirs used a fixed front-facing US image (retaining the familiar view of US images on a US console) [27], while ours allowed the user to choose an arbitrary 3D vantage point to view the 2D image from. In both systems, virtual representations of tracked surgical instruments supplemented the 3D view of the surgical scene, with the intention to improve the visualization and success rates of the needle-insertion. The lack of improvement in our system and that proposed by Ameri et al. is likely due to the decoupling between the clinician’s motor and visual fields when the US console or a monitor was used as the visualization device.

In contrast, the use of HMD, which delivered an MR guidance system using a first-person immersion vantage point, significantly improved the outcome of CVC insertion. The CVC insertion success rate and accuracy (measured as the distance between the final tip location to the center line of the vessel and from the vessel wall) were improved when compared to US-only guidance. These results emphasize the importance of using an HMD to ensure a coherent visual and motor field during needle guidance. This is achieved in the HMD by bringing the needle guidance information directly into the line-of-sight of the clinician. While the monitor-based system would be more readily integrated into a clinical workflow, our results suggest there are benefits for using an HMD for needle guidance, promoting the continued pursuit of research related to the use of HMDs in similar clinical scenarios.

The US-only case resulted in a 68% success rate for the expert cohort, which is low despite the simplicity of the phantom. We believe there were several factors affecting the outcome of this guidance approach. During analysis, it was observed that clinicians may not use the US-guidance information effectively to follow the needle tip throughout the insertion, as in most US-only cases the US image remained fairly stationary. In addition, we observed a high reliance on muscle memory as the user would align the needle with the center of the probe and in one swift motion perform the insertion based on intuition. The user would verify that the needle was in the US image based on its reflection. However, in many cases the users captured the shaft instead of the tip of the needle, leading the user to erroneously believe that they had performed the insertion successfully. Additionally, as this is the approach with which they are most familiar, there is a potential that they were overconfident in the accuracy of their needle placement given the simplicity of the phantom.

In terms of complication rates, there was no significant difference in the rates of CA puncture between these three modes of visualization implemented for our experiment, as the overall rates of CA puncture were low. One expert and two novices punctured the CA, and all punctures occurred under US-guidance. The observed low complication rate of CA puncture is likely due to the simplicity of our anthropomorphic neck phantom as the IJV and CA had a simple orientation with limited overlap as it was laterally positioned to the IJV (Figure 2). Additional experiments using a range of neck phantoms with diverse anatomical variations are planned future projects. As depicted in Figure 2, the appearance and configuration of the neck vasculature are variable. The utilization of US to guide needle-insertion in the SAX/out-of-plane approach has the inherent inability to track the position of needle tip once it is traversed beyond the US image plane. In a realistic clinical scenario, the inadvertent posterior IJV wall punctures could result in damage to critical anatomical structures adjacent to the IJV, including CA, but this was not the case using our phantom. While our study did not show that the first-person immersive MR system had a significant effect on the rates of CA puncture, the HMD system has indeed resulted in higher rates of successful insertions as the participants positioned the needle closer to the center of the vessel with fewer posterior wall punctures than US-only guidance.

The feasibility of using an HMD in a clinical setting is a critical concern for using the first-person immersive MR for needle guidance. Results of the questionnaire responses suggested that the first-person immersive MR HMD system may be more feasible when used for training rather than for clinical deployment, as on average these clinicians ranked the clinician viability of the system a 4.35/10 compared to a 7.04/10 for usefulness for training. Most clinicians indicated that they would consider using advanced visual guidance on an ad-hoc basis, with the belief that the more complicated cases could benefit from the advantages offered by the advanced visualization system. We are currently employing the HTC VIVE Pro as a virtual-reality display device even though it has stereo cameras and can be used as an augmented reality video-pass-through display. Incorporation of the stereo camera feeds for visual guidance may facilitate the clinical acceptability of this first-person immersive technology as the clinician would be able to visualize the guidance information while maintaining a direct view of the real surrounding environment. However, such an approach is not feasible with current devices as the stereo camera image resolution and fidelity are low. Alternatively, mixed-reality optical-see-through HMD devices such as the Microsoft Hololens could be used, but extensive evaluation of the accuracy tracking (i.e., camera hand-eye calibration) and careful integration with optical- or magnetic-tracking systems would be required.

Our results, in conjunction with those presented by Ameri et al. [27], suggest that visualizing the 3D spatial relationship of surgical instruments using a 2D display does not provide sufficient guidance for a user to perceive the 3D context of the surgical scene, regardless of whether the US image is viewed in a front-facing or an oblique perspective. In contrast, a 2D monitor-based MR system has been developed and successfully applied to focal liver tumor ablation [37], prostate brachytherapy [38], and breast biopsy [39], suggesting there is a potential pathway to implement a clinically feasible monitor-based guidance system for CVC insertion. Developing and incorporating advanced 3D visualization to improve 3D perception using a 2D monitor is a planned future project. Alternatively, a tablet-based display, which could be situated closer to the surgical site, could facilitate greater coherence of the visual and motor fields.

## 5. Conclusions

This paper describes the development of a mixed-reality guidance system for central venous catheterization that provides the 3D spatial relationship between the streaming US video and magnetically tracked surgical needle and US transducer with respect to patient anatomy using either a 2D monitor or a head-mounted display. The objective of this work was to compare the performance of medical professionals in a needle-insertion task under three different mode of US visualization: (1) the standard US-only visualization displayed on the US console, (2) a 3D MR visualization on a 2D monitor, and (3) a 3D MR visualization on an HMD, thus providing a first-person perspective view. We recruited 31 expert clinicians and 20 medical students who performed needle-insertions with a tracked hypodermic needle into an anthropomorphic neck phantom using each of these three systems. Compared to the standard US-only visualization guidance used clinically, our data suggest that the use of an HMD system significantly improves the successful needle-insertion rate for both expert and novice cohorts. When using the HMD system, the final needle tip locations were significantly closer to the center of the vessels than the vessel walls, indicating that this mode also significantly improves the accuracy of the needle placement. From these findings, we conclude that the ability of the operator to interpret the 3D spatial relationship between the surgical instruments and patient needle is imperative to successful CVC needle-insertion, and that the use of a fist-person perspective mixed-reality system can facilitate alignment of the clinicians’ visual and motor fields.

## Figures and Tables

**Table 1:** Post-hoc test results following repeated measures ANOVA analysis for the distance from the final needle tip position to the closest point on the center line of the vessel for both experts and novices.

Experts		Mean Difference (mm)	t	p_*bonf*_
US Only	Monitor	−0.5	−0.5	1.00
	HMD	2.7	3.1	0.008 **
Monitor	HMD	3.1	3.7	0.001 **
**Novices**		**Mean Difference (mm)**	**t**	**p_*bonf*_**
US Only	Monitor	0.5	0.300	1.00
	HMD	6.4	4.0	0.001 **
Monitor	HMD	6.0	3.6	0.002 **

** *p* < 0.01. *Note. p*-value and confidence intervals adjusted for comparing a family of 3 estimates (confidence intervals corrected using the Bonferroni method).

**Table 2:** Post-hoc test results following repeated measures ANOVA analysis for the distance from the final needle tip position to the closest point on the vessel wall for both experts and novices.

Experts		Mean Difference (mm)	t	p_*bonf*_
US Only	Monitor	0.2	−1.8	1.0
	HMD	−2.0	−4.0	0.04 *
Monitor	HMD	−2.3	−2.8	0.02 *
**Novice**		**Mean Difference (mm)**	**t**	**p_*bonf*_**
US Only	Monitor	−0.07	−0.05	1.0
	HMD	−5.0	−8.7	0.005 **
Monitor	HMD	−4.9	−3.3	0.006 **

** *p* < 0.01, * *p* < 0.05. *Note. p*-value and confidence intervals adjusted for comparing a family of 3 estimates (confidence intervals corrected using the Bonferroni method).

**Table 3:** Summary of the success rates by system and the associated statistics. Systems with significant differences in success rates (*p* < 0.05) are denoted with *.

Success Rate (%)	US-Only	Monitor	HMD	$\chi^{2}$	*p*
Experts	67.7% *	64.5%	93.5% *	8.034	0.018 *
Medical Students	25.0% *	25.0%	70.0% *	13.71	0.001 *

**Table 4:** Summary of insertion time and number of users who punctured the CA under each mode of visualization.

		System	
**Insertion Time**	**US-Only**	**Monitor**	**HMD**
Experts	11.46±6.42	9.94±10.45	7.82±4.41
Novices	11.48±7.45	12.23±7.26	11.49±6.47
**CA punctures**	**US-Only**	**Monitor**	**HMD**
Experts	1	0	0
Novices	2	0	0

**Table 5:** User questionnaire results scored out of 10, with 10 being the best score.

Question	Average Score
How viable is the HMD to use in the OR?	4.35 ± 2.82
If the system was clinically available how often would you use it?	4.75 ± 2.7
How useful do you think the HMD system would be for training US-guided CVC?	7.04 ± 2.25
How was the comfort associated with using the HMD?	7.64 ± 2.62

## Data Availability

Data available on request due to restrictions.

## Abbreviations

The following abbreviations are used in this manuscript:

2D	Two-Dimentional
3D	Three-Dimentional
AR	Augmented Reality
AV	Augmented Virtuality
CA	Carotid Artery
CVC	Central Venous Catheterization
DoF	Degrees of Freedom
FoV	Field of View
IJV	Internal Jugular Vein
LAX	Long Axis
HMD	Head Mounted Display
MR	Mixed Reality
MTS	Magnetic Tracking System
PVAc	polyvinyl alcohol cryogel
SAX	Short Axis
SoS	Speed of Sound
US	Ultrasound
VPT	Video Pass Through
VR	Virtual Reality
